# Ethnobotanical knowledge on indigenous fruits in Ohangwena and Oshikoto regions in Northern Namibia

**DOI:** 10.1186/1746-4269-9-34

**Published:** 2013-05-22

**Authors:** Ahmad Cheikhyoussef, Werner Embashu

**Affiliations:** 1 Indigenous Knowledge System Technology (IKST) Food & Beverage Program, Science, Technology and Innovation Division, Multidisciplinary Research Centre (MRC), University of Namibia, Private Bag 13301, Windhoek, Namibia

**Keywords:** Ethnobotanical knowledge, Ethnoveterinary knowledge, Indigenous fruits, Namibia

## Abstract

**Background:**

Indigenous communities in Namibia possess a rich indigenous knowledge expressed within many practices of these communities. Indigenous wild edible fruits are available along the Namibian 13 regions of which it forms a rich source of vitamins, fibres, minerals and a heterogeneous collection of bioactive compounds referred to as phytochemicals for indigenous people’s diet. The aim of this study was to record the different IKS practices on the indigenous fruit trees in Ohangwena and Oshikoto regions of Namibia.

**Methods:**

An ethnobotanical survey was undertaken to collect information from local communities from 23-29 October 2011. Data was collected through the use of questionnaires and personal interviews during field trips in the Ohangwena and Oshikoto regions. A total of 65 respondents were interviewed; 54%; women, 38%; men and 8%; both in group interviews.

**Results:**

The majority of the people interviewed were in their thirty’s, with the youngest being 18 years old and the oldest being 98 years old. Forty three plant specimens were collected from the two regions; these specimens belong to 20 genera and 25 species. Regarding to the indigenous knowledge; 87%; of the respondents indicated that their knowledge on indigenous fruits was learnt mainly through their parent. Indigenous people’s perception on declining indigenous fruits revealed that 56.3%; of the respondents reported that indigenous fruits were declining. Only a 42.2%; indicated that the indigenous fruits populations are increasing. Regarding to the management practices to improve the production of these indigenous fruit trees; 38.6%; reported that there are some efforts on management practices; on the other hand 61.4%; reported there are no management practices on the indigenous fruit trees in their areas. Four species were found to be the most frequently used and mentioned fruits which need to be given high preference in terms of conservation are: *Berchemia discolor*, *Hyphaene petersiana*, *Sclerocarya birrea* and *Diospyros mespiliformis*. The following diseases and ailments have been reported to be treated by the indigenous fruit trees which include: toothache, diarrhoea, cough, tonsillitis, burns, skin allergy, stomach ache, snake bit, constipation, etc. 28%; of the respondents mentioned an ethno veterinary use(s) of these fruits, e.g. the use of the *Ziziphus mucronata* roots to treat diarrhoea in cattle, the bark of eembe (*Berchemia discolor*) to treat calf weakness.

**Conclusions:**

The local communities in Oshikoto and Ohangwena regions have relatively good knowledge and practices regarding the indigenous fruit. This study enhances our understanding on the indigenous fruit in Namibia and their uses by local communities.

## Background

Research into Indigenous Knowledge System Technology (IKST) has been increasing dramatically in Namibia during the last five years. The common knowledge to a particular community or people living together in a certain area, generated by their own and their ancestors experience is generally referred to as indigenous knowledge (IK) to that specific society [1], this term has been described as the local knowledge that is unique to a given culture or society [2]. This indigenous knowledge is regarded as valuable and considered as the local people’s capital [3,4], and if not preserved, it may be lost forever to society. With the development of the knowledge economy, knowledge has become one of the most important resources for social progress and economic development [5].

Great emphasis has been recently laid on the role of traditional food in the health and nutritional status of the people. Most of traditional food have a good proportion of some nutrients and can make a significant contribution to daily nutrient intake, especially for those of low social classes. Additionally, traditional food constitutes an essential aspect of cultural heritage and they are highly regarded by the community [6]. Despite lack of scientific knowledge, many local peoples understand the benefits of indigenous food in maintaining their culture and in health promotion. Traditional food is being studied for scientific identification, nutritional composition and cultural food use; however, there is still much to be done and be learned [7].

Indigenous fruits play essential roles in food security, human health and nutrition, and economic welfare of rural communities in the developing world [8]. The importance of wild fruits in the diet depends to a large extent on availability of domestic fruits. Most wild fruits are consumed in spring and summer, and this consumption been associated with potential health benefits [9]. Indigenous fruit trees have been given only limited attention, both in research and development, though they are important in the diet [10]. These indigenous fruit trees play important roles in the lives of rural peoples in Namibia [11], Botswana [12], Zimbabwe [13], Ghana [14], Benin [15,16], and Ethiopia [17,18]. The consumption of indigenous fruits in Ohangwena and Oshikoto forms a daily life practice and it is gaining an increasing interest. This consumption gives an important contribution to local communities’ health and welfare [19]. In addition, indigenous fruits contain higher amounts of nutrients and bioactive compounds [20]. Therefore, the aim of this study was to record different IKS practices on the indigenous fruit trees in selected constituencies in Ohangwena and Oshikoto regions.

## Methods

### Study areas and sampling

**Figure 1:**
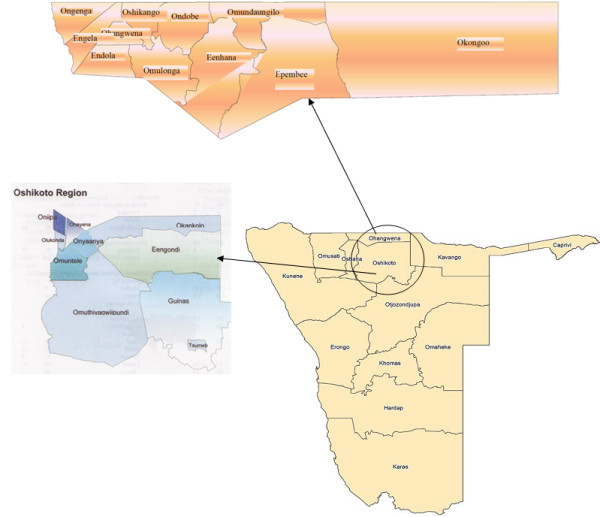
Map of the study area showing Namibia and the two studied regions.

The study was carried out in the Ohangwena and Oshikoto region. Ohangwena and Oshikoto are two of the Namibian 13 political regions (Figure 1). Ohangwena is the most densely populated region in Namibia. Ohangwena Region has a population of 245,446 of which 99%; live in rural areas. Ohangwena Region has the highest population density at 23.20 persons per square kilometer [21]. The region is divided into 11 constituencies, namely: Endola, Ongenga, Engela, Oshikango, Ohangwena, Ondobe, Eenhana, Epembe, Omundaungilo, Okongo and the newly established Omulonga constituency. With an area of 10582 km^2^ the region stretch along the Angolan boarder to the North, Omusati and Oshana regions to the West, Oshikoto to the South and Okavango to the East. The annual temperature range between 23-34°C and the annual rainfall varies between 480 mm and 600 mm. This survey was conducted in Eenhana and Okongo constituencies based on their geographical location and their known flora. Oshikoto has a subtropical climate, with very hot summers and mild winters. The average maximum temperature lies at 29.7°C, while the average minimum temperature is 14.4°C. The average rainfall is 555 mm per annum [22]. The Oshikoto region was selected on the basis that it is inhabited by people from different ethnic groups: the San, Owambo, Damara/Nama, and Ova Herero. It has ten constituencies: Oniipa, Onyaanya, Onayena, Olukonda and Omuntele, Okankolo, Engodi, Genius, Omuthiya and Tsumeb constituencies. It was chosen as a study site because it could provide data useful for design of proper data collection instruments for a country-wide baseline study for the indigenous fruits in Namibia. This survey was conducted in Oniipa and Onayena constituencies based on previous survey conducted in 2008 [23]. An official permit to collect plant specimens was obtained from the Ministry of Environment and Tourism.

Before research activities were initiated, regional council and town council were both visited to inform them about the field trip research background and the research activities at the Multidisciplinary Research Centre (MRC), University of Namibia. Official permission letters from the Regional Council Offices were sent to the respective village’s headman. Before the interviews start; the team leader gives an overview of the objective of the research field trip to the local community in some villages. Participants from each village were selected upon recommendations from the headman based on their knowledge of their own people.

### Ethnobotanical survey

A structured questionnaire was used for interviews. Interviews were conducted in the local language; Oshiwambo. Only a few were done in English, where the people who can understand the language. Interviews were done individually and group interviews were taken with exceptional cases were people were found gathered at one place. Plant specimens were collected and preserved in a plant press. The GPS coordinates for the collected specimens were recorded as well. Each of the plants received a voucher specimen number and voucher specimen/collection forms were completed for the plants that were collected, digital photos were also taken. Later on the specimens were sent to the National Botanical Research Institute (NBRI) for scientific identification. The questionnaires focused on the investigation of local name of the species, which parts are used, traditional product(s) made from the tree, management practice, abundance, traditional fruits health and social value(s) and how people perceived the decline/increase of the species in time.

### Data analysis

After listing, the indigenous fruits plant species were ranked based on frequency of being mentioned as most important. The number of times each species was cited as most important amongst the listed five species served as priority index [23]. Species cited between 3-9 times were assigned moderate priority; 10-20 times, high priority and 21 or more times were highest priority criteria. Only plants cited as most important for three or more times were considered in the ranking [23]. The presence index for the high and highest priority species in the survey area were also calculated.

## Results and discussion

### Ethnobotanical uses of indigenous fruit

A total of 65 interviews were conducted in total; with 40 interviews in Ohangwena region and 25 in Oshikoto region. The majority of the people interviewed were in their thirty’s, with the youngest being 18 years old and the oldest being 98 years old. Within the 65 respondents; 54%; women, 38%; men and 8%; were both in group interviews. Most of the respondents 87%; indicated that their knowledge on indigenous fruits was learnt mainly through their parents expect for a few have reported that their knowledge were learnt from some people in their villages 11%; and schools 2%;. All respondents reported that the indigenous fruits and their products have social values such as it is presented to guests at special ceremonies, weddings, etc. In addition to their social values; health values have been reported also especially when someone having flu, it can be given as a juice (Marula juice). Some respondents have reported that indigenous fruits can be used as first aid before seeking treatment in hospital for some disease and ailment such as cough and flu. Some people depend on these fruits and their by-products to get an income, for example distilled liquor from Eembe (*Berchermia discolor*), Palm fruits (common practice in Oshikoto region). All respondents reported that the traditional beer and wines are the main products made from these fruits (Table 1). On the ethnobotanical knowledge and ethno-pharmacological uses of the indigenous fruit, it is reported that the leaves of *Hyphaene petersiana* and the roots of *Diospyros lycioides* are used as toothbrush. The following diseases and ailments have been reported to be treated: toothache, diarrhoea, cough, tonsillitis, burns, skin allergy, stomach ache, heartburn, snake bit, constipation, etc. Many products have been reported to be produced from monkey orange, eembe and marula [24], also oils from marula and *Ximenia* ssp. This result is in agreement with Motlhanka et al. [12] who reported on the traditional fruits derived products in Botswana (morula beer, morula jelly, morula oil) from Marula (*Sclerocarya birrea)* and Ximenia oil from *Ximenia caffra* and *Ximenia americana*. Some respondents have reported on adding these fruits (*Berchemia discolor* and *Grewia* sp.) to their local porridges and others have reported that they prefer to dry them and eat it during times of food shortages like *Hyphaene petersiana*. These results are in agreement with Feyssa et al. [17,18] who reported on the nutritional value of *Berchemia discolor* and its different uses by local communities in Ethiopia and on the contributions of the indigenous fruits (*Berchemia discolor*, *Debera glabra*, *Grewia* sp., *Ximenia americana*, and *Ziziphus spina-christi*) to food and healthcare security of the semiarid people in Ethiopia.

**Table 1:** Indigenous fruits species, plant part(s), by-products and their use for special ceremonies, for its nutrition by local communities in the studies areas in Ohangwena and Oshikoto region, Namibia

**Scientific name**	**Local name**	**Plant part(s)**	By-products*	Uses and significance*
*Ancylanthos rubiginosus*	Omumbu	Fruits	Wine, food	Generate income
		Leaves	NR	Medicinal
*Annona stenophylla*	Omutyaalale	Fruits	Food	NR
*Berchemia discolor*	Omuye	Fruits	Wine, food	Generate income
*Boscia albitrunca*	Omunkunzi	Roots	Flavouring agent	Added to sour milk
		Leaves	NR	Medicinal
*Dialium engleranum*	Omufimba	Fruits	Food	Generate income
*Diospyros mespiliformis*	Omwandi	Fruits	Food additive	To increase nutritional value of infant porridge
*Diospyros lycioides*	Oshimumu	Roots	Tooth sticks	Generate income
*Ficus sycomorus*	Omukwiyu	Leaves, Twigs	NR	Medicinal
*Grewia avellana*	Omukopakopa	Fruits	Beer, food	Generate income
*Grewia flavescens*	Omushe	Fruits	Beer, food	Generate income
*Grewia schinzii*	Omushe	Fruits	Beer, food	Generate income
*Guibourtia coleosperma*	Omushii	Fruits	Food additive	To increase nutritional value of infant porridge
*Hyphaene petersiana*	Omulunga	Fruits	Wine	Generate income
		Leaves	Weaving baskets	
*Parinari capensis*	Omukokofi	Roots	NR	Medicinal
*Pygmaeothamnus zeyheri*	Unknown	N.R.	NR	NR
*Salacia luebbertii*	Okandongondongo	Fruits	Food	Generate income
		Roots	NR	Medicinal
*Schinziophyton rautanenii*	Omunkete	Fruits	Wine	Generate income
		Kernels	Food Thickener, oil	
*Sclerocarya birrea*	Omugongo	Fruits	Wine, beer, food	Generate income
		Kernels	Oil	Wedding, social visits
*Searsia tenuinervis*	Omupombo	Leaves	NR	Medicinal
*Spirostachys africana*	Omuhongo	Twigs	NR	Medicinal
*Strychnos pungens*	Omupwaka	Fruits	Beer, food	Generate income
*Strychnos spinosa*	Omuuni	Fruits	Beer, food	Generate income
*Ximenia americana*	Oshikukulu	Fruits	Beer, food	Generate income
*Ximenia caffra*	Oshimbyupeke	Kernels	Oil	Skin and hair softeners
*Ziziphus mucronata*	Omukekete	Fruits	Wine, food	Medicinal

**: NR; Not Reported by respondents.

### Plant parts used for diseases treatment

Sixty five respondents reported 25 different indigenous fruit trees, 43 plant specimens were collected of which 24 are from Ohangwena and 19 from Oshikoto region, these specimens belong to 20 genera and 25 species (Table 2). In this survey, only 6 indigenous fruit species have not been reported to treat any ailments or diseases (Table2). Many common diseases and ailments such as constipation, diarrhoea, headache, dough, flu, toothache, nose bleeding, skin allergy, tonsillitis, heartburn, wounds, snake bite, measles, stomachache, ear infection, epilepsy, sore fingers and unstable pregnancy have been reported to be treated by indigenous fruit trees (Table 2). These results are in agreement with Busia [25] who reported that common ailments (headaches or coughs) in Africa are treated at the household level because such diseases are considered to be connected with natural causes and hence their symptoms. Various studies have reported on the ethnobotanical uses of indigenous fruits such as ackee (*Blighia sapida* K.D. Koenig) in the treatment of fever, burns, anemia, vomiting, malaria, eye problems and snakebite [15], Marula (*Sclerocarya birrea*) in treating malaria, stomach-ache, diarrheoa, haemorrhoids cough and diabetes [20] and African star apple (*Chrysophyllum albidum* G. Don) in the treatment of malaria, anaemia, ulcer, haemorrhoids, smallpox, asthma, cough, dental decay, yellow fever and avitaminosis [16]. Many of the plants based prescriptions by traditional healers or knowledge holders can be considered as general health tonics which can be used to treat general weakness and unspecified ailments [26].

**Table 2:** Ethno-pharmacological knowledge on the indigenous fruit used by local communities in the studied areas in Ohangwena and Oshikoto region, Namibia

**Scientific name**	**Local name**	**Family**	**Voucher number**	Ailment or diseases*	Plant parts used *
*Ancylanthos rubiginosus* Desf.	Omumbu	Rubiaceae	IKSTF 022	Toothache	Roots
				Tonsillitis	Leaves
				Heartburn	Twig
*Annona stenophylla* Engl. & Diels subsp. nana (Exell) N. Robson	Omutyaalale	Annonaceae	IKSTF 019	NR	NR
*Berchemia discolor* (Klotzsch) Hemsl.	Omuye	Rhamnaceae	IKSTF 001	Flue & cold	Leaves
				Skin itching	Bark
				Nose bleeding	Bark
*Boscia albitrunca* (Burch.)Gilg & Benedict	Omunkunzi	Capparaceae	IKSTF 039	Constipation	Leaves
				Headache	Roots
*Dialium engleranum* Henriq.	Omufimba	Fabaceae	IKSTF 009	NR	NR
*Diospyros mespiliformis* Hochst. ex A.DC.	Omwandi	Ebenaceae	IKSTF 007	NR	NR
*Diospyros lycioides* Desf.	Oshimumu	Ebenaceae	IKSTF 031	Toothache	Roots
*Ficus sycomorus* L. subsp. *gnaphalocarpa* (Miq.) C.C.Berg	Omukwiyu	Moraceae	IKSTF 044	Constipation	Stem
*Grewia avellana* Hiern	Omukopakopa	Malvaceae	IKSTF 021	Diarrhoea	Roots
*Grewia flavescens* Juss*.* var. *flavescens*	Omushe	Malvaceae	IKSTF 011	Cough	Leaves
				Diarrhoea	Roots
*Grewia schinzii* K. Schum.	Omushe	Malvaceae	IKSTF 027	Heartburn	Fruits
*Guibourtia coleosperma* (Benth.) J.Léonard	Omushii	Fabaceae	IKSTF 006	Cough	Leaves
*Hyphaene petersiana* Klotzsch ex Mart	Omulunga	Arecaceae	IKSTF 037	Dry Cough	Kernel, root
				Wounds	Leaves
*Parinari capensis* Harv. subsp. *capensis*	Omukokofi	Chrysobalanaceae	IKSTF 042	Snake bites	Roots
*Pygmaeothamnus zeyheri* (Sond.)Robyns var. *zeyheri*	Unknown	Rubiaceae	IKSTF 020	NR	NR
*Salacia luebbertii* Loes.	Okandongondongo	Celastraceae	IKSTF 024	Wounds	Roots
*Schinziophyton rautanenii* (Schinz) Radcl.-Sm.	Omunkete	Euphorbiaceae	IKSTF 018	Measles	Leaves
*Sclerocarya birrea* (A. Rich.)Hochst*.* subsp. *birrea*	Omugongo	Anacardiaceae	IKSTF 023	Toothache	Roots
				Tonsillitis	Leaves
				Heartburns	Sticks
				Cough	Branch
				Ear infection Epilepsy	Kernel (oil) Bark
*Searsia tenuinervis* (Engl.) Moffett	Omupombo	Anacardiaceae	IKSTF 016	NR	NR
*Spirostachys africana* Sond*.*	Omuhongo	Euphorbiaceae	IKSTF 013	Snake bites	Twig
*Strychnos pungens* Soler.	Omupwaka	Loganiaceae	IKSTF 008	Stomachache	Roots
*Strychnos spinosa* Lam.	Omuuni	Loganiaceae	IKSTF 010	NR	NR
*Ximenia americana* L. var. *microphylla* Welw. ex Oliv.	Oshikukulu	Olacaceae	IKSTF 015	Constipation	Leaves
*Ximenia caffra* Sond. var. *caffra*	Oshimbyupeke	Olacaceae	IKSTF 005	Stomachache	Roots
				Unstable pregnancy	Roots
*Ziziphus mucronata* Willd.	Omukekete	Rhamnaceae	IKSTF 002	Skin allergy and rush	Leaves Roots
				Sore fingers	Leaves

*: NR; Not Reported by respondents.

**Figure 2:**
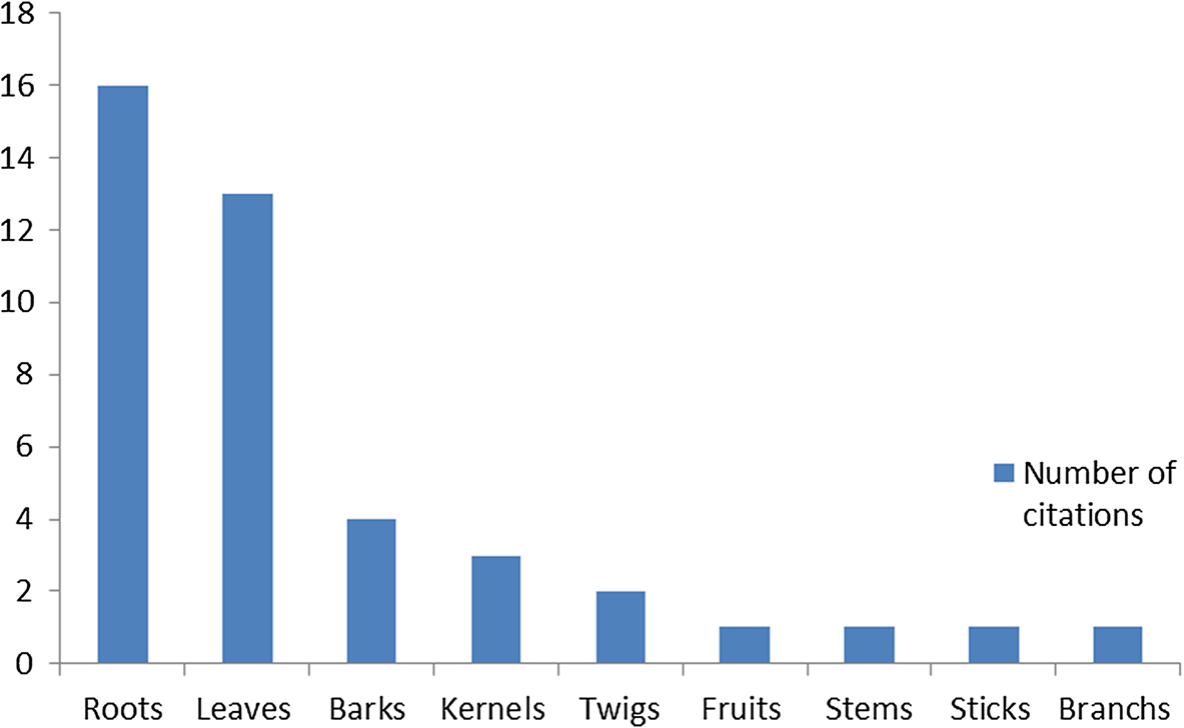
Status of use for indigenous fruit parts.

Regarding to the indigenous trees parts used by local peoples in Ohangwena and Oshikoto regions; roots and leaves where the most frequently used for the treatment of diseases (Figure 2). The result is in agreement with Teklehaymanot [27] who reported that the roots are used in the preparations of traditional remedies. It is also in agreement with Panghal et al. [28] and Tabuti et al. [29] who reported on uses of leaves as most used parts among the Saperas community of India and Nakapiripirit, Pallisa, Kanungu, and Mukono districts of Uganda. It is also in agreement with results from an ethnobotanical survey reported by Cheikhyoussef et al. [26] whereby roots reported to be the most frequently used for the treatment of diseases by traditional healers.

### Ethnoveterinary knowledge

In regard to the priority ethnoveterinary fruit uses; seven species were considered important and were used to treat six ailments or diseases that commonly infect livestock in these two regions in Namibia (Table 3). 28%; of the respondents have mentioned at least one ethnoveterinary use of indigenous fruit trees, e.g. the use of the *Ziziphus mucronata* or *Grewia avellana* roots to treat diarrhoea in cattle, the bark of Omuye (*Berchemia discolor*) to treat calf weakness. The roots and leaves of *Grewia flavescens var. flavescens* were used to treat the retained placentia in cattle (Table 3). Snake bites in livestock were treated by using the roots of *Parinari capensis* (Table 3). Roots were found to be the most used plant part to treat different ailments and diseases in animals, followed by leaves, bark and kernel (Figure 2). This practice is of importance for the indigenous people in Oshikoto and Ohangwena regions whereby most of them depend on their livestock on daily basis. The high cost involved and inaccessibility to conventional remedies have helped local communities to maintain traditional treatment practices in these countries and fostered research on this subject [30]. As is known traditional animal healthcare system (also known as ethnoveterinary medicine) has a very long history of domestication of animals for human needs [31-33].

**Table 3:** Plant species used for ethnoveterinary purposes in the studies areas in Ohangwena and Oshikoto regions, Namibia

**Voucher number**	**Local name**	**Scientific name**	**Family**	**Ailment or diseases**	**Plant parts used**
IKSTF 001	Omuye	*Berchemia discolor* (Klotzsch) Hemsl.	Rhamnaceae	Calf weakness	Bark
IKSTF 002	Omukekete	*Ziziphus mucronata* Willd.	Rhamnaceae	Cattle diarrhoea	Roots
IKSTF 011	Omushe	*Grewia flavescens* Juss. var. *flavescens*	Malvaceae	Cattle retained placentia	Leaves & roots
IKSTF 015	Oshikukulu	*Ximenia americana* L. var. *microphylla* Welw. ex Oliv.	Olacaceae	Goat swollen eye	Leaves
IKSTF 021	Omukopakopa	*Grewia avellana* Hiern	Malvaceae	Cattle diarrhoea	Roots
IKSTF 037	Omulunga	*Hyphaene petersiana* Klotzsch ex Mart.	Arecaceae	Dog lung disease	Kernel
IKSTF 042	Omukokofi	*Parinari capensis* Harv. subsp. *capensis*	Chrysobalanaceae	Snake bites in livestock	Roots

To the best of our knowledge, this is the first study to report on the ethnoveterinary knowledge from Namibia. Veterinary medicine is an important practice in developing countries where conventional remedies for animal health care are inaccessible or unaffordable to indigenous people [33]. According to the United Nations Food and Agricultural Organization (FAO), the lack of drugs to treat diseases and infections results in losses of 30-35%; in the breeding sector of many developing countries, where poor animal health remains the major constraint to increased production [34]. Ethnoveterinary practices can improve animal healthcare and hence enhanced living standards of the local communities [35,36]. Ethnoveterinary knowledge is part of the body of traditional knowledge which is increasingly becoming more relevant to conservation biology, public health policies, sustainable management of natural resources and biological prospection [36,37].

### Informant consensus

**Figure 3:**
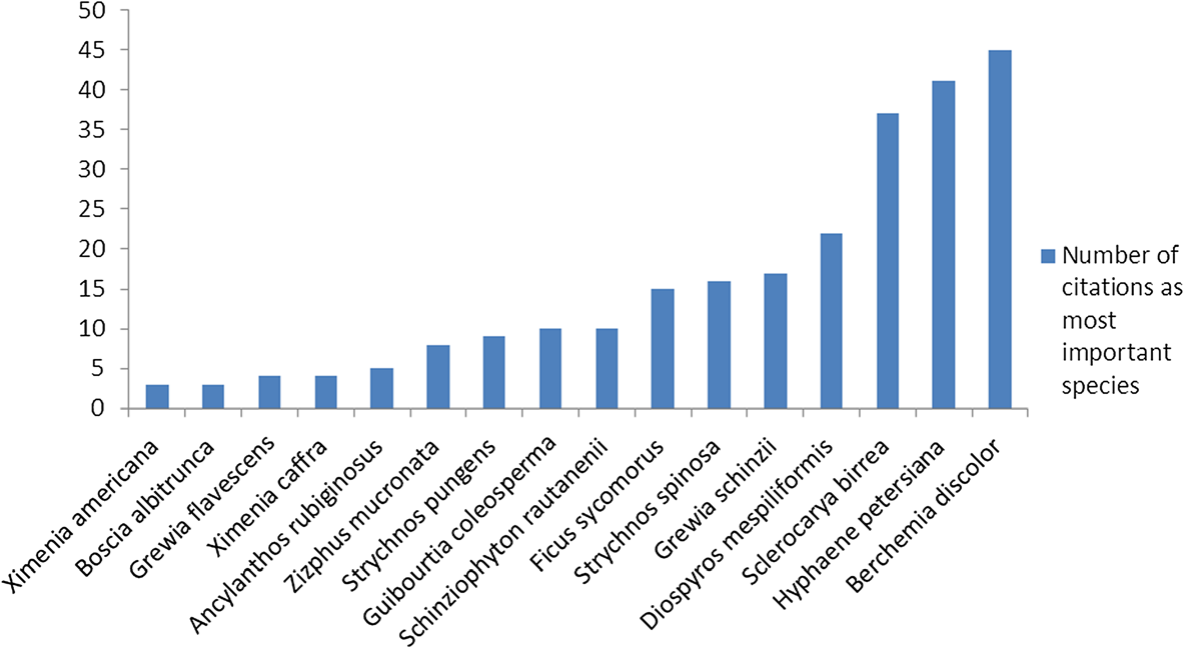
Number of times indigenous fruit species are ranked as most important in Ohangwena and Oshikoto regions, Namibia (N = 16; 3-9 citations moderate priority species, 10-20 citations-high priority species, ≥ 21- highest priority species).

In term of priority; this study identified 16 species which were recognized as priority indigenous fruit trees for the local communities in the studied areas. Seven species were classified as moderate, 5 species with high priority and 4 were the highest priority (Figure 3). These four tree species which need to be given high preference in terms of conservation are: *Berchemia discolor, Hyphaene petersiana*, *Sclerocarya birrea* and *Diospyros mespiliformis*. This high preference rank is known to be an important index in identifying plants of potentially high conservation concern [24,38]. These species have been utilized differently in Namibia of which *Sclerocarya birrea* has received more attention in term of domestication and cultivars for fruit production and because of its by-products verities. The other three species (*Berchemia discolor*, *Hyphaene petersiana* and *Diospyros mespiliformis*) mainly used to produce traditional beer and often eaten fresh or dry. These fruits have multiple uses as vital component of Namibia natural vegetation, environment protection and socio-cultural aspects.

**Figure 4:**
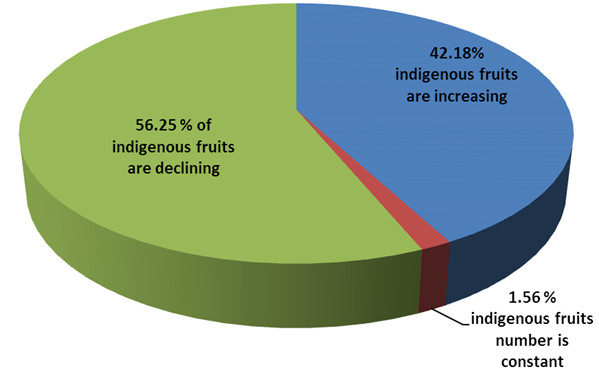
Knowledge index regarding indigenous fruit among local people in Ohangwena and Oshikoto regions, Namibia (figures refer to percentage of respondents).

**Figure 5:**
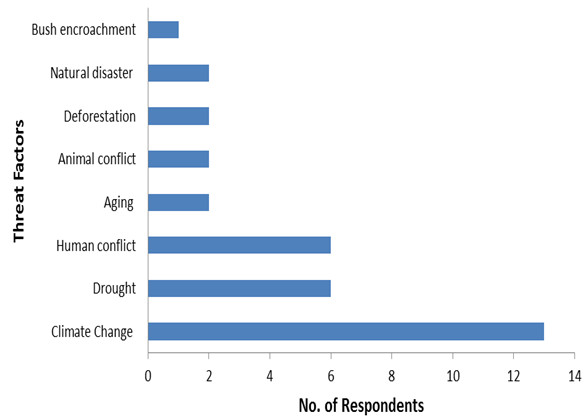
Factors responsible of the decline of indigenous fruit in Ohangwena and Oshikoto regions in Namibia.

**Figure 6:**
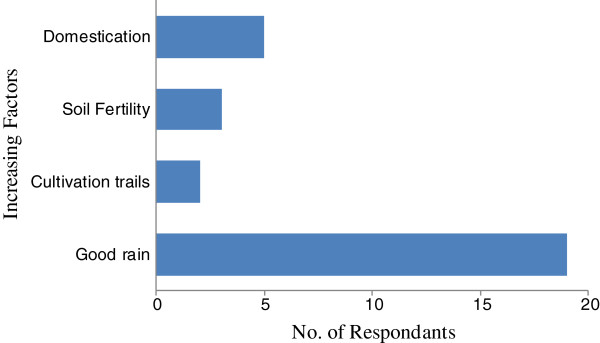
Factors responsible of the increasing population of indigenous fruit in Ohangwena and Oshikoto regions in Namibia.

Results regarding indigenous people perception on declining local indigenous fruits revealed that 56.3%; of the respondents reported that indigenous fruits were declining (Figure 4). Some of the reasons and factors contributing to this phenomena (Figure 5) are mainly the climate changes which include; drought, cold and freezing, natural disasters, deforestification and heavy rain. Other factors are human and animal conflict such as housing, fire wood and farming. Only a 42.2%; indicated that the indigenous fruits populations are increasing (Figure 6), while only 1.6%; recognized the fact that indigenous fruit trees number is constant. During the field survey some of these respondents remarked that as long as it rain, indigenous fruits cannot be threatened. Other reasons on the increasing status are the fertility of the soil, cultivation trails, protection and domestication efforts from local communities and government ministries (Figure 6). This study shows the necessity to increase the public awareness and to build capacity in the local communities of these two regions regarding its indigenous fruit trees with regard to their conservation status, domestication process and propagation methods. This is in agreement with Njoroge et al. [24] who suggested propagation and domestication methods to local communities in Mwingi district in Kenya to protect medicinal plants to optimize the sustainability of natural resources [24].

**Figure 7:**
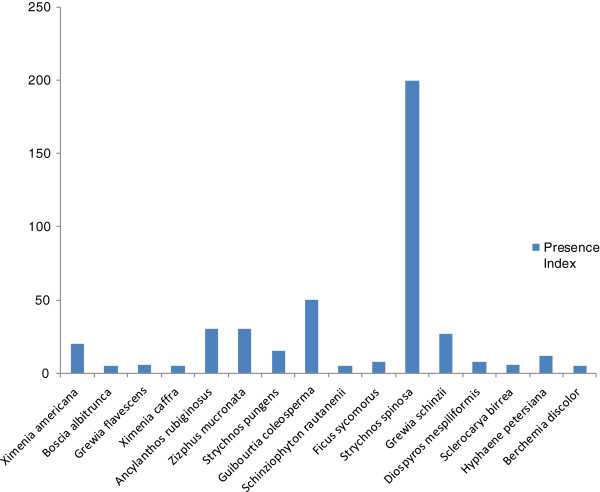
Presence index regarding indigenous fruit among local people in Ohangwena and Oshikoto regions in Namibia (figures refer to number indigenous fruits trees in their household area or their farmland).

**Figure 8:**
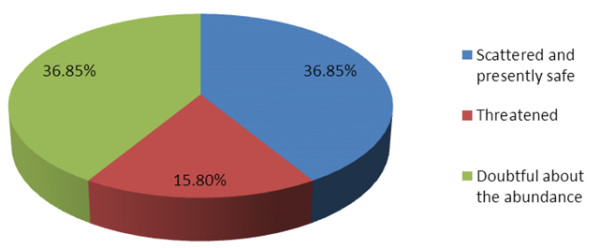
The abundance status of the indigenous fruit in Ohangwena and Oshikoto regions, Namibia (figures refer to percentage of respondents).

In terms of presence index; presence index results showed that these indigenous peoples in the studied areas have at least 3-4 (in average) indigenous fruits trees in their household area or their farmland with highest number of 200 and the lowest number of 1 fruit tree (Figure 7). On the abundance of the indigenous fruits; most of the respondents indicated that most of the indigenous fruit are scattered but presently safe (36.9%;) on the other hand some of the interviewees (15.8%;) say the fruit trees in their villages are threatened and 36.9%; interviewees are doubtful about its abundance (Figure 8).

### Traditional management practices

Regarding to the management practices to improve the production of these indigenous fruit trees; 38.6%; reported that there are some efforts on management practices; on the other hand 61.4%; reported there are no management practices on the indigenous fruit trees in their areas. Domestication (in domo cultivation) of indigenous fruit trees could be a good alternative to overcome their overexploitation from the wild [39]. This strategy is widely adopted in Europe, China and India for many medicinal plants [40]. In Namibia, this process gave promising results on Marula tree (Sclerocarya *birrea* subsp. *caffra)* in North Central Region of Namibia [41-43]. The selection of Marula cultivars for fruit production has considered the needs of traditional beer/wine markets and new markets for fruit juices, flavorings, liqueurs, etc. Marula cultivars selection led to a considerable increasing in uniformity and productivity in the product, and has provided an incentive for farmers to plant marula trees in their farming systems [44].

The similarities with the results from other indigenous fruits, which are being domesticated in some other parts of Africa such as West Africa using a Participatory Domestication approach, suggest that a similar participatory strategy to marula domestication should be taken to ensure that the local communities are the beneficiaries [44]. The participatory domestication of indigenous fruits has been proposed as an appropriate means to alleviate poverty [45] and could also have positive benefits on the environment [40].

In addition to Marula, farmers and local communities are encouraged to plant other useful trees, so diversifying the farming system with likely benefits on sustainability, through the creation of an agro-ecological succession culminating in a mature or climax phase [46]. Additionally, such developments would also help to sustain some of the traditional values of marula in the culture, for example the ‘first fruits’ ceremony and the traditional role of marula beer in the society [40].

The establishment of a database for indigenous fruits and their ethnobotanical uses would reduce the chances of this valuable information to disappear, whilst also contributing to the awareness of the conservation importance of these fruits. Sharing indigenous knowledge within and across communities will also help enhancing cross-cultural understanding and promote the cultural dimension of development especially the optimum utilisation of natural resources by the local communities in Namibia.

## Conclusions

A total of 65 interviews were conducted with 40 interviews in Ohangwena region and 25 in Oshikoto region. Forty three plant specimens belong to 20 genera and 25 species were collected for scientific identification and further investigations. Thirty six indigenous fruit were used for healing humans and 7 species been used to treat animals by the local communities in the studied regions. Roots were found to be the most used plant part in the treatment for both human and animals. The most important species found in this study based on the priority index are: *Berchemia discolor*, *Hyphaene petersiana*, *Sclerocarya birrea* and *Diospyros mespiliformis.* Regarding the perception on declining indigenous fruits revealed that 56.3%; of the respondents reported that indigenous fruits were declining meanwhile 42.2%; reported on increasing and 1.6%; are stable. There were no significant differences between the selected studied areas in the two regions in terms of the indigenous knowledge on indigenous fruits. Management practices are needed to be consolidated and promoted to cover more species in the studied areas and domestication process to be one of the suggested solutions with promising results on Marula (*Sclerocarya birrea*) in Eenhana constituency*.*

## Competing interest

The authors declare that they have no competing interests.

## Authors’ contributions

WE had interviewed the indigenous peoples in Oshikoto and Ohangwena region; he has performed a preliminary statistical analysis (Presence and priority indexes). AC has written the article, organized the research data and carried out the statistical analysis. Both authors read and approved the final manuscript.
